# DEEP DIVES INTO STATE POLICY: LOCATING LEVERS TO IMPROVE CARE FOR ADULTS AGING ALONE

**DOI:** 10.1093/geroni/igae098.0199

**Published:** 2024-12-31

**Authors:** Jane Lowers

**Affiliations:** Emory University, Atlanta, Georgia, United States

## Abstract

The Health and Aging Policy Fellowship facilitates fellows’ exposure to the intricacies of policy development and implementation in a variety of settings. My HAPF placement at ADvancing States provided an opportunity to investigate how individual states define “family” in regard to caregiving for older adults, both through regulations and Medcaid waivers. For state aging and disability directors, this analysis offered insight into how state policies aligned with federal policies, including the Older Americans Act, and opportunities to adapt other states’ efforts to support unpaid caregiving networks. As a researcher, this fellowship work provided insight into the types of evidence needed to advocate for equitable services and supports for adults aging without biological family. This presentation will provide an overview of HAPF placement focused on state-level policies.

